# Incompatibility of resazurin to detect viable cells in lignin biomaterials: a cautionary study

**DOI:** 10.1186/s13104-026-07667-z

**Published:** 2026-02-13

**Authors:** T. Meghana, Swekcha, Bharath Raja Guru, Anoushka Mukharya, Srinivas Mutalik, Abhayraj S. Joshi

**Affiliations:** 1 Department of Biotherapeutics Research, Manipal Academy of Higher Education, Manipal, Karnataka 576104 India; 2 Department of Biotechnology, Manipal Institute of Technology, Manipal Academy of Higher Education, Manipal, Karnataka 576104 India; 3 Department of Pharmaceutics, Manipal College of Pharmaceutical Sciences, Manipal Academy of Higher Education, Manipal, Karnataka 576104 India

**Keywords:** Lignin, Alkali lignin, Methacrylated lignin, Resazurin, AlamarBlue™, Cell viability assay, MTT assay, A549 cells

## Abstract

**Objective:**

The nanoparticles prepared from lignin polymer and its methacrylated derivative have gained significant attention in the biomedical field owing to their superior biosafety profile. Here, this study aims towards the synthesis of the methacrylated lignin nanoparticles (MLNPs) using the nanoprecipitation method and understanding their biocompatibility in A549 cells using the resazurin assay.

**Results:**

In our study, the nanoprecipitation method yielded a stable suspension of MLNPs having sub-200 nm size. When incubated with A549 cells, initially, these nanoparticles showed cytotoxicity and reduced biocompatibility from the well-known resazurin assay. However, careful microscopic examination of the cells showed healthy and well-adhered A549 cells with normal cobblestone-like epithelial morphology. Hence, we performed a detailed analysis of MLNPs to understand their interaction with resazurin with respect to concentration and time while keeping pristine lignin nanoparticles (LNPs) as a control. We found that LNPs and MLNPs interfered with resazurin assay, leading to false-positive results. Our study highlights a significant drawback in the use of resazurin assay for these nanoparticles and emphasizes the critical need for alternative approaches to evaluate their biocompatibility. In an attempt to find alternative, conventional MTT assay offered highly reproducible biocompatibility results without any interference during evaluating the cytotoxicity of these nanoparticles.

**Graphical Abstract:**

**Supplementary Information:**

The online version contains supplementary material available at 10.1186/s13104-026-07667-z.

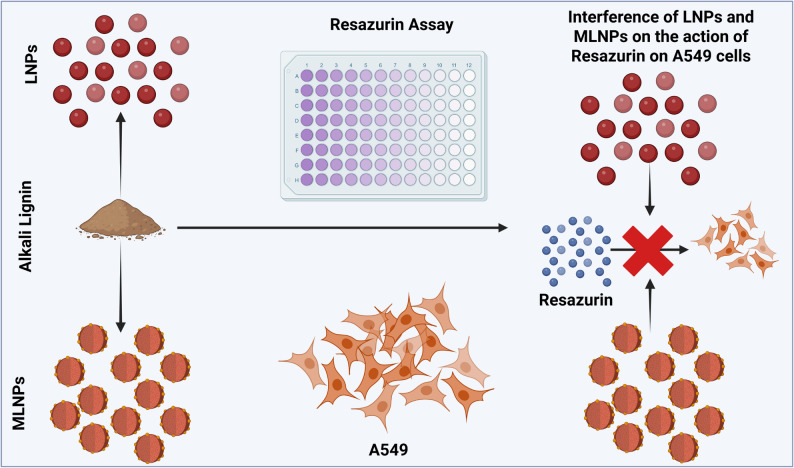

## Introduction

Lignin is a fundamental secondary metabolite in plant cells, synthesized by the phenylalanine/tyrosine metabolic pathway [1]. After cellulose and hemicellulose, lignin is the most abundant natural polymer, comprising 30% of the carbon content in the biosphere [2]. The lignin polymer exhibits various properties such as excellent biocompatibility, ease of chemical modification [3], inherent antioxidant and antimicrobial properties [4], which can be exploited for developing novel biomaterials. Till date, lignin biomaterials such as lignin nanoparticles and lignin hydrogels have been employed for various biomedical applications, such as drug delivery [5], tissue engineering [6], and biosensing [7]. Recent reports show that lignin nanoparticles can be prepared using nanoprecipitation, solvent fractionation, emulsion-solvent evaporation, electrospinning, self-assembly, and various other methods, resulting in spherical nanoparticles (< 200 nm) with enhanced surface reactivity [8–13]. Irrespective of any biomedical application, the lignin nanoparticles must fulfil the first and foremost criterion of biocompatibility. The cell viability assay proves in vitro biocompatibility and yields key preliminary information about the basic understanding of how cells respond to new biomaterials. To do this, researchers use a variety of cell viability assays, which generally fall into three technical categories: colorimetric, fluorometric, and luminescence assays indicating how healthy and/or active cells are via different detection methods [14].

Colorimetric assays detect color changes linked to metabolic activity of the cell or membrane integrity, whereas the fluorometric assays pick up fluorescent signals from cellular metabolites. On the other hand, the luminescent assays measure ATP levels, an indicator of cellular energetics and activity [15]. The most commonly used assays include 3-(4,5-dimethylthiazol-2-yl)-2,5-diphenyltetrazolium bromide assay (MTT), 2,3-bis-(2-methoxy-4-nitro-5-sulfophenyl)-2 H-tetrazolium-5-carboxanilide assay (XTT), Cell Counting Kit-8 (CCK-8), Lactate dehydrogenase assay (LDH), and Resazurin (commercially available as alamarBlue™). Among these assays, the Resazurin assay is based on a redox reaction in which resazurin, a blue non-fluorescent dye, is reduced by viable cells into resorufin, a pink and fluorescent metabolite [16]. Resazurin offers distinct advantages over other dyes in terms of non-toxic and cytocompatible nature, high sensitivity as well as reproducibility, capability of high-throughput screening, and applicability in both mammalian and microbial cells [17, 18].

In this report, we intend to highlight that, irrespective of the great advantages of highly sensitive resazurin dye, it is incapable of accurately determining cell viability for lignin nanoparticles. A recent report suggests the applicability of resazurin in the determination of cell viability of lignin nanoparticles intended for cytotoxic action, and the authors showed that those nanoparticles were cytocompatible till a concentration of less than 0.1 mg/ml [19]. In this contradictory report, for the first time, we have shown that lignin interacts with resazurin by some unknown mechanism that could lead to erroneous conclusions about cell viability in the presence of these nanoparticles. With this report, we strongly advise using an alternative cell viability assay, preferably conventional MTT or other similar assay for lignin nanoparticles for reproducible and reliable results. At present, it is difficult to pinpoint the exact mechanism of interference of lignin with resazurin assay and the question of the mechanism of lignin interaction with resazurin still remains open-ended. In this manuscript, we have shown that LNPs and MLNPs do not interfere with MTT assay and hence, MTT serves as a more robust and reliable alternative for evaluating the cytotoxicity of lignin-based nanoparticles.

## Results and discussion

**Fig. 1:**
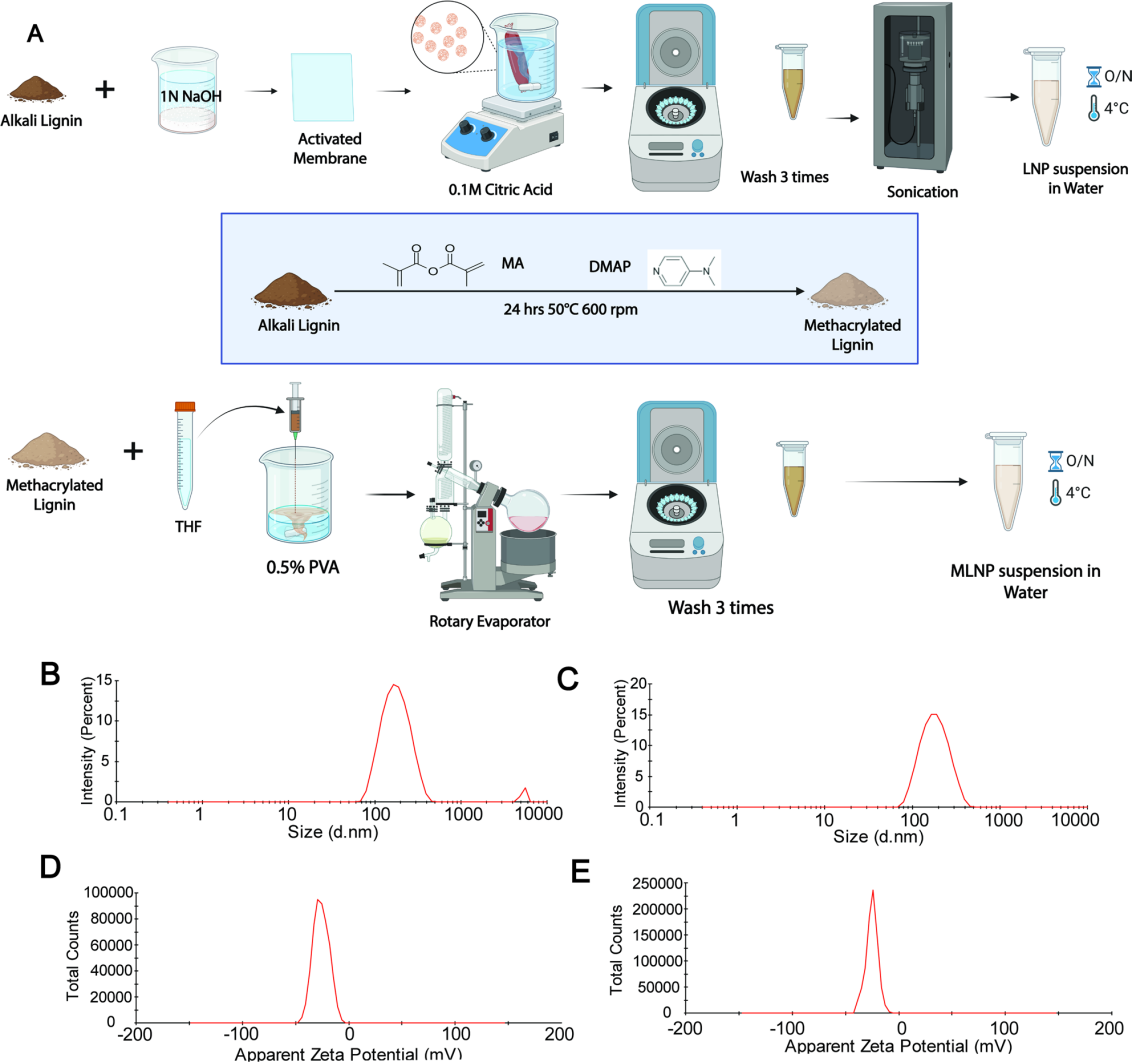
**A** Schematics represent the synthesis process for LNPs (upper row) and MLNPs (lower row). **B**,** C** Hydrodynamic size distributions for LNPs and MLNPs which were measured to be 182.3 ± 1.05 nm and 176.3 ± 3.29 nm, respectively. **D**,** E** Zeta potential profiles for LNPs and MLNPs which were measured to be -26 ± 1.44 mV and − 24.8 ± 0.7 mV, respectively

To explore the drug delivery potential of ML polymer, we started our work with the aim of establishing cytocompatibility of MLNPs in the A549 lung cancer cell line. Hence, we first established protocols for the synthesis of (i) LNPs, (ii) ML, and (iii) MLNPs (Fig. 1A).

### Characterization of LNPs and MLNPs using the DLS method

The DLS analysis of LNPs and MLNPs revealed their unimodal size distribution within the range of 100–300 nm (Fig. 1, B&C). The average particle sizes for LNPs and MLNPs were 182.3 ± 1.83 nm and 176.3 ± 5.7 nm, respectively. This suggests that both methods of synthesis of LNPs yielded a relatively uniform nanoparticle population with negligible aggregation (Fig. 1, B&C). It has been previously shown that such size uniformity below 200 nm and monodispersity (PDI of LNP: 0.332 ± 0.03 and PDI of MLNP: 0.168 ± 0.02) are essential for better cellular uptake, biodistribution, and effective delivery of cargo, as nanoparticles below 200 nm exhibit optimal biological interactions [20, 21]. Next, the zeta potentials of LNPs and MLNPs were − 26 ± 2.5 mV and − 24.5 ± 1.2 mV, respectively. Negative zeta potentials within the range of approximately − 20 mV to -30 mV suggest that the nanoparticles possess good electrostatic stability in the suspension owing to the presence of the surface functional groups present on lignin and its methacrylated derivative. The negatively charged surfaces generate electrostatic repulsion among individual nanoparticles when they come into proximity, thereby preventing aggregation and sustaining Brownian motion [22]. Moreover, zeta potential is recognized as a critical physicochemical parameter governing nanoparticle–cell interactions (nano–bio interactions) and plays a decisive role in modulating endocytic uptake and intracellular trafficking of these nanoparticles [23]. Overall, the DLS profiles of both nanoparticles established successful formation of the stable nanoparticles.

### Cell viability assay and effect of nanoparticles on resazurin reduction

**Fig. 2:**
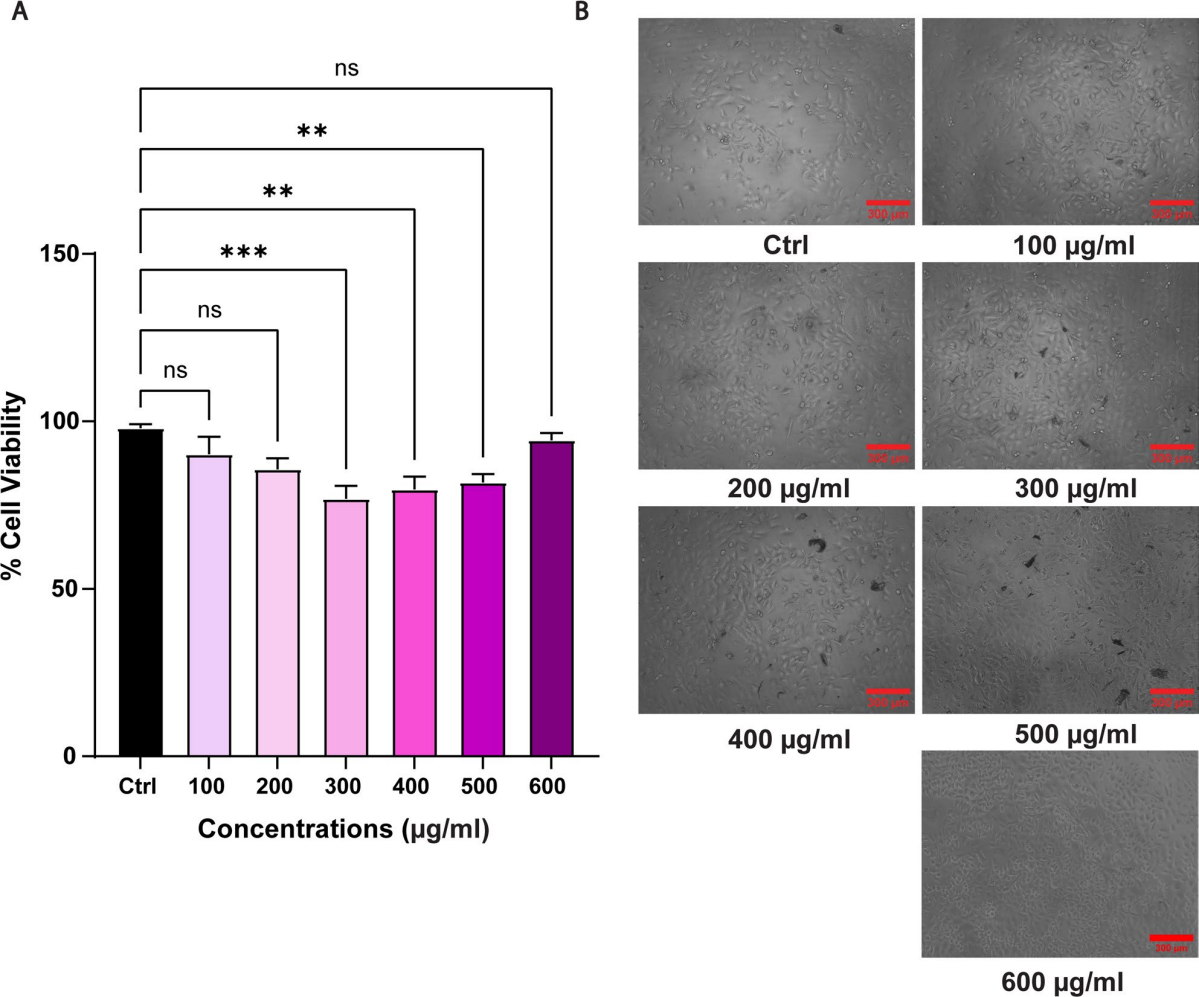
**A** The bar graph show viability of A549 cells determined using resazurin after incubating cells with 100–600 µg/ml of MLNPs. Each bar represents mean ± std. error of 6 replicates (*n* = 6). The asterisks in the bar graphs represent significantly different observations ( One-way ANOVA test, * p-value < 0.05, **p-value < 0.01). **B** Bright-field microscopy images show morphology of A549 cells in absence and presence of MLNP (Scale bar = 300 μm)

As ML has been employed for developing drug delivery systems and hydrogels [24–26], we started our research work to explore the potential of MLNPs for spatiotemporal delivery of anticancer phytochemicals. Since, for clinical utility and regulatory approval of any drug delivery system, biocompatibility is one of the important aspects, using the A549 cell line, we first determined the cell viability of MLNPs. To our surprise, as compared to control, MLNPs showed significant cell death (One way ANOVA, *p*-value < 0.05) in the concentration range of 100–600 µg/ml. The average cell viability reduced from 92.89 ± 4.89% to 80.30 ± 9.57% for 100 µg/ml to 300 µg/ml, showing concentration-dependent effect (One-way ANOVA, *p*-value < 0.05) that got diminished at higher concentrations (Fig. 2A) ultimately resulting in erratic pattern in the cell viability. However, the microscopic examination of control and nanoparticle-treated A549 cells (Fig. 2B) revealed no apparent signs of cytotoxicity. The morphological analysis of the cells showed an intact biomembrane along with cobblestone-like epithelial morphology. This indicated that MLNPs did not exert cytotoxic effects on the cells, irrespective of the change in fluorescence intensity of resazurin dye after conversion via reduction reaction. This observation also hinted towards the possibility of the reduction of resazurin into resorufin due to interaction with both MLNPs and cells. It has been shown in the literature that nanoparticles such as carbon nanoparticles can alter the reduction of resazurin dye [27, 28]. Hence, based on our results, we presumed two possibilities. First, ML directly reduces resazurin to resorufin [27], or alternatively, the presence of MA, a byproduct of methacrylation reaction, that makes the solution acidic, may convert resazurin to resorufin [29].

**Fig. 3:**
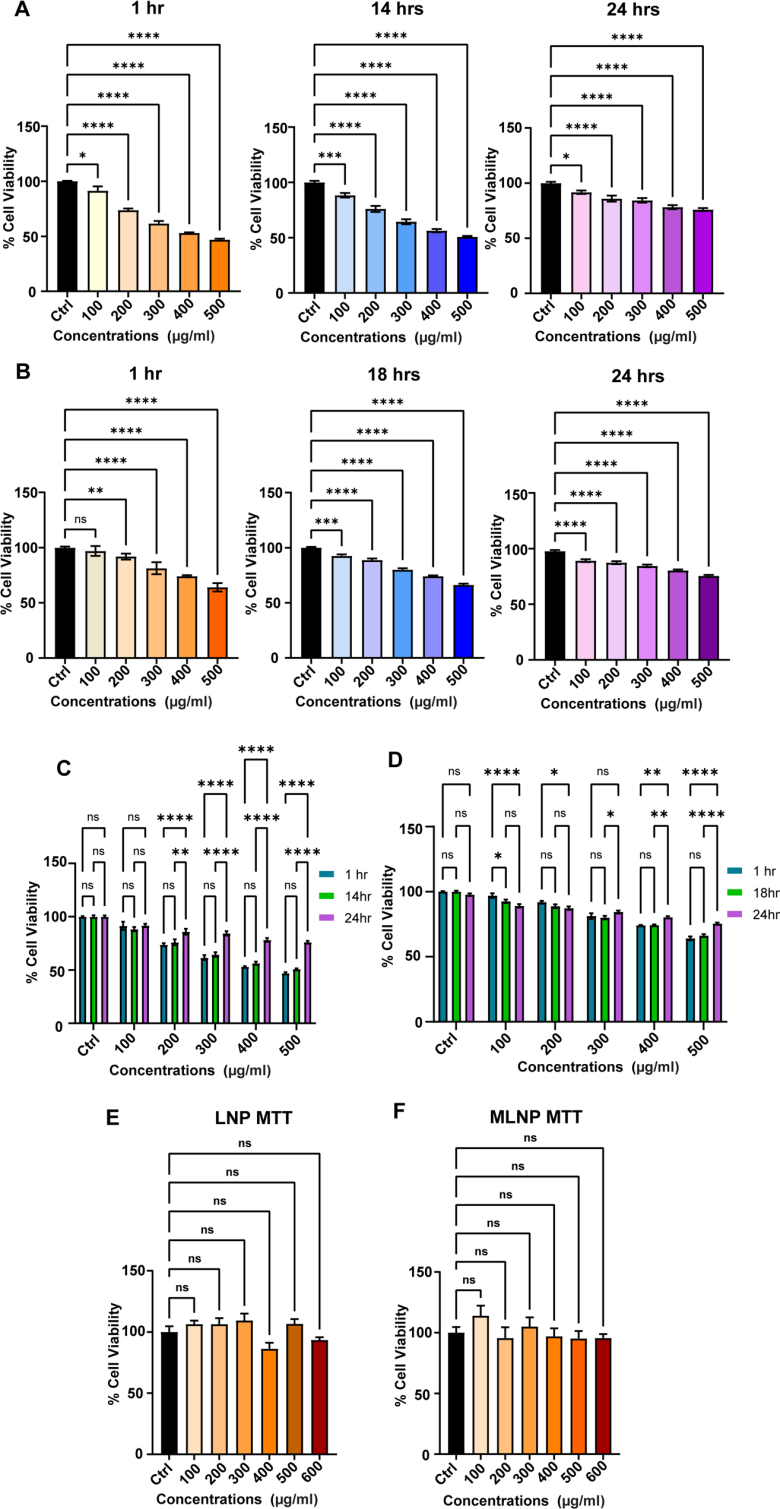
**A**,** B** The bar graphs show fluorescence intensity of resazurin after reduction to resorufin in presence of LNPs and MLNPs in concentration range of 100–500 µg/ml. Each bar represents mean ± std. error of 6 replicates (*n* = 6). The asterisks in the bar graphs represent significantly different observations when compared to the control group (One-way ANOVA test, * p-value < 0.05, **p-value < 0.01, *** p-value < 0.001, **** p-value < 0.0001). **C**,** D** The bar graphs show statistical analysis of fluorescence intensity of resorufin in presence of LNPs and MLNPs across three different timepoints (1 h, 14 h (for LNPs), 18 h (for MLNPs), and 24 h). The asterisks in the bar graphs represent significantly different observations when compared with respect to time (One-way ANOVA test, * p-value < 0.05, **p-value < 0.01, *** p-value < 0.001, **** p-value < 0.0001). **E**,** F** The bar graphs shows the cytotoxicity analysis of LNP and MLNP by MTT assay, where each bar represents mean ± std. error of 6 replicates (*n* = 6)

But, in our experiments, we performed extensive washing with an excess of deionized water after methacrylation. The pH of the final wash was in the range of 6–7 which nullified the possibility of the presence of MA in the mixture. To rule out the first possibility, we synthesized LNPs that are devoid of its methacrylated derivative and MA. Then, we incubated both LNPs and MLNPs with resazurin solution in separate well plates in the absence of A549 cells to confirm the dye reduction (Fig. 3, A-D). As suspected, both the LNPs and MLNPs showed statistically significant changes in the fluorescence intensities (One-way ANOVA, *p*-value < 0.05) at all tested concentrations (100–600 µg/ml), confirming the interference of pristine and methacrylated lignin polymer in dye reduction. Additionally, in literature, the delayed resazurin metabolism has been reported in viable as well as dead hepatocytes [30]. Hence, we tried to determine the interference of pristine and methacrylated lignin polymer in dye reduction at different time points (1 h, 14 h (for LNPs), 18 h (for MLNPs), and 24 h). Across these timepoints, the disparity was the same in both LNPs and MLNPs (Fig. 3, A-D). Moreover, the comparison for each concentration for the given time point yielded variable and inconsistent changes in resorufin fluorescence (Fig. 3, C&D). Unlike our previous experiment (Fig. 2A), the MLNPs after 24 h showed dose-dependent changes in fluorescence intensity (Fig. 3B, 24 h) even at higher concentration (> 300 µg/ml) that again led to erratic pattern. Under given circumstances, we thought to deviate a little for accurate detection of resorufin fluorescence. Hence, we collected only supernatant medium from the wells which had resorufin and excluded the nanoparticles and A549 cells present at the bottom of the well. We assumed that the detection of the resorufin fluorescence in supernatant might omit the interference of LNPs or MLNPs. However, we observed similar interference in the fluorescence intensity (Figure S1, A&B) showing dose-dependent reduction in fluorescence intensity at lower concentrations and rise at higher concentrations. We observed similar erratic pattern in the change of fluorescence intensity in the supernatant when LNPs and MLNPs were incubated with dye in absence of A549 cells (Figure S1, C&D). It also confirmed that this disparity is not a random phenomenon owing to the physical presence of nanoparticles and scattering and/or reflection caused by them. In this experiment, when compared to control, all treatment groups showed statistically significant changes in cell viability (One-way ANOVA, *p*-value < 0.05) (Figure S1). This trend strongly suggests that lignin interferes with the resazurin dye, converts it to resorufin by some unknown mechanism, and shows false-positive results.

### MTT as an alternate assay to resazurin

Since LNPs and MLNPs showed interference in resazurin assay, we resorted to a commonly used cell viability dye – MTT. The MTT assay relies on the mitochondrial dehydrogenase mediated intracellular reduction of a water-soluble MTT dye into its insoluble purple colored formazan derivative. This formazan derivative is retained within cells that can be subsequently solubilized in DMSO and quantified by measuring absorbance at 570 nm [31]. Upon performing the MTT assay, we observed that the A549 cell viability remained statistically similar as compared to the control (One-way ANOVA, *p*-value > 0.05) (Fig. 3, E&F). This concluded that LNPs as well as MLNPs showed excellent biocompatibility in A549 cells. This observation further corroborated our previous microscopic morphological analysis of the cells (Fig. 2B). Similar experiment when performed without cells, we observed no adsorption of MTT onto lignin nanoparticles or interference with the assay output, indicating that the nanoparticles do not contribute to or interfere in the measured light intensity (Figure S2). Since the insoluble formazan product is separated from the nanoparticle suspension during washing and solubilization, we assumed that the LNPs and MLNPs are less likely to influence the readout under standard conditions. Therefore, for reliable cytotoxicity assessment of alkali lignin polymer based nanomaterials, we recommend replacing resazurin assay with the conventional MTT assay with appropriate control groups .

## Limitations

From our presented work, we conclude that the synthesis of pristine LNPs and MLNPs via dialysis and nanoprecipitation methods yielded monodisperse particles below 200 nm. This fulfilled one of the ideal characteristics of nanotherapeutics intended for biomedical applications. While checking the cytocompatibility, the MLNPs exhibited strong interference with the resazurin assay. Similar interference from LNPs as well as MLNPs in the absence of cells confirmed poor sensitivity of the resazurin assay for lignin biomaterials. In alternate method, we observed that conventional MTT assay is immune to such interference making it a reliable option in our study.

At the moment, we believe that there could be at least 3 probable reasons for our negative results:

1. Resazurin gets reduced to resorufin by lignin in the solution, thus resulting in erroneous and erratic results.
2. It is also possible that lignin dissolution and/or degradation may lead to the release of phenolic aldehydes like vanillin or syringaldehyde, which may play a role in resazurin reduction.
3. Adsorption of resazurin on nanoparticles and its subsequent reduction on the surface of the nanoparticles.

Understanding the exact mechanism needs a careful experimental plan and an appropriate technique to determine the interaction and kinetics of resazurin reduction in presence of lignin. Nonetheless, this interference study underscores the necessity of orthogonal cytotoxicity assessments when testing lignin nanomaterials along with morphological analysis of cells.

## Supplementary Information

Below is the link to the electronic supplementary material.

Supplementary Material 1.

## Data Availability

All data generated or analyzed during this study are included in this published article [and its supporting information files].

## Abbreviations

DLS: Dynamic light scattering
DMEM: Dulbecco’s modified eagle medium
DMAP: 4-(Dimethylamino)pyridine
DPBS: Dulbecco’s phosphate-buffered saline
LNP: Pristine lignin nanoparticle
MA: Methacrylic anhydride
ML: Methacrylated lignin
MLNP: Methacrylated lignin nanoparticle
MTT: 3-(4,5-dimethylthiazol-2-yl)-2,5-diphenyltetrazolium bromide
PDI: Poly dispersity index
PVA: Poly (vinyl alcohol)
THF: Tetrahydrofuran
