# Supplementary material for: Incompatibility of resazurin to detect viable cells in lignin biomaterials: a cautionary study
Source: BMC Res Notes. 2026 Feb 13;19:119. doi: 10.1186/s13104-026-07667-z (PMC13005528; doi:10.1186/s13104-026-07667-z)
Supplement: Supplementary file 1 — Supplementary Material 1. [file 13104_2026_7667_MOESM1_ESM.docx]

**Supporting Information**

**Incompatibility of Resazurin to Detect Viable Cells in Lignin Biomaterials – A Cautionary Study**

**Meghana T., Swekcha, Bharath Raj Guru, Anoushka Mukharya, Srinivas Mutalik, Abhayraj S. Joshi**

**Materials:**

The alkali lignin (Cat. No. 471003), resazurin sodium salt (Cat. No. R7017), methacrylic anhydride (MA) (Cat. No. 276685), poly (vinyl alcohol) (Cat. No. P8136) (MW: 30000-70000 Da), 4-(Dimethylamino)pyridine (DMAP) (Cat. No. 107700), and dimethyl sulfoxide (DMSO) (Cat. No. 1.07046.0521) were purchased from Sigma-Aldrich, India. Dulbecco's phosphate-buffered saline (DPBS) (Cat. No. 21600-010), Dulbecco’s modified eagle medium (DMEM) high glucose (Cat. No. 12800-017), penicillin streptomycin (Pen Strep) (Cat. No. 15140-122), and trypsin (Cat. No. 25200056) were procured from Thermo Fisher Scientific Inc., India. 3-(4,5-Dimethyl-2-thiazolyl)-2,5-diphenyl-2H-tetrazolium bromide (MTT) (Cat. No. MB186-1G) and fetal bovine serum (Cat. No. RM1112) were purchased from HiMedia Laboratories LLC, India. Human lung adenocarcinoma cell line (A549 cells) was purchased from NCCS, Pune, India. Tetrahydrofuran (THF) (Cat. No. 92562) was purchased from Sisco Research Laboratories Pvt. Ltd. (SRL), India.

# **Methods:**

1. **Synthesis of pristine lignin nanoparticles (LNPs) using hydrophilic alkali lignin:**

The LNPs were synthesized using activated dialysis membrane (Molecular weight cutoff 12000-14000 Da). Lignin was dissolved in 1M NaOH solution at a concentration of 1.5 mg/ml and introduced into the dialysis membrane. Then it was dialyzed against 40 ml of 10% acetic acid on a magnetic stirrer at 300 RPM for 48 h at room temperature. The resultant suspension was then transferred into a 1.5 ml microcentrifuge tube and washed twice using distilled water at room temperature by performing centrifugation for 10 min at 8000 RPM. Then, using a Sonics Probe Sonicator, the resultant suspension was sonicated for 1 min (3 s ON and 2 s OFF, at 30% amplitude). The obtained LNPs were then stored at 4 °C for further experiments.

1. **Synthesis of methacrylated lignin (ML) polymer:**

Alkali lignin, originally soluble in aqueous solvents, was modified using MA and DMAP to prepare ML as per previously published protocol (1). Briefly, 1 g of alkali lignin was first dissolved in 22 ml of distilled water. To this solution, 1 ml of MA and 48 mg of DMAP were added. This mixture was kept on a magnetic stirrer at 600 RPM for about 48 h at 60 ºC. The resultant mixture was washed thrice with distilled water by performing centrifugation at 7000 RPM for 10 min at room temperature. The mixture was then lyophilized for 48 h to yield dry ML.

1. **Synthesis of methacrylated lignin nanoparticles (MLNPs):**

The MLNPs were synthesized using the nanoprecipitation method. 10 mg of ML was first dissolved in 1 ml of THF. MLNPs were synthesized by introducing this lignin solution into 0.5% PVA solution (1:10 Organic phase: Aqueous phase ratio) under constant magnetic stirring at 600 RPM. The mixture was stirred for 10 min, and then the organic phase was removed by rotary vacuum evaporation. The nanoparticle pellet was collected by centrifugation at 13000 RPM for 30 min and washed thrice with distilled water. The final pellet was resuspended in 1 ml of distilled water and stored at 4 ºC for further experiments.

1. **Characterization of nanoparticles using the dynamic light scattering (DLS) technique:**

The 100 µl of LNPs and MLNPs suspensions were diluted to 1 ml with distilled water (1:9 ratio) and were subjected to particle size and zeta potential measurements using Malvern Zetasizer ZS90. All measurements were performed in triplicate, and results were reported as mean ± standard error.

1. **Cell culture:**

*In vitro* cell culture study was performed using A549 cells. All experiments were performed with cells from passage numbers 75-79 using high-glucose DMEM supplemented with 10 % FBS and 1X Pen Strep. All the experiments were performed at 37 °C in a humidified incubator provided with 5% CO_2_.

1. **Cell Viability using Resazurin Assay:**

A549 cell line was grown and maintained in high-glucose DMEM supplemented with 10% FBS until 75% confluency. Then, the cells were harvested by trypsinization and seeded in a 96-well plate at a starting seeding density of 7500 cells/well. After allowing them to adhere and grow for 24 h, the cells were treated with LNPs and MLNPs at concentrations ranging from 100 - 600 µg/ml. After 24 h incubation, the old medium was replaced with 100 µl of new DMEM containing 30 µg/ml resazurin dye (Stock concentration of resazurin: 10 mg/ml). The cells were then incubated with resazurin for 1 h at 37 °C. After incubation, the fluorescence intensities were measured at an emission wavelength of 590 nm (excitation wavelength: 540 nm) using a BIOTEK microplate reader. The data were reported in terms of % cell viability relative to control untreated cells and as mean ± standard error of 6 replicates (n=6). To check the effect of nanoparticles on cellular morphology, the images were captured before resazurin addition using an Invitrogen EVOS M5000 brightfield microscope at 10X magnification. Alternatively, to reduce the interference of LNPs and MLNPs in plate reader readout owing to scattering and/or reflection, we performed a similar experiment. Before measuring the fluorescence intensity, the 100 µl of supernatant from each well was transferred to new 96 well plate to take readings separately.

1. **Resazurin Assay of LNPs and MLNPs:**

To evaluate the direct interaction of the dye with nanoparticles in the absence of cells, an identical protocol was followed. Briefly, the LNPs and MLNPs were aliquoted into a 96-well plate at concentrations ranging from 100 – 600 µg/ml, with each well containing a total of 200 µl of nanoparticle suspension in complete DMEM high-glucose medium. Subsequently, resazurin dye was added to each well at a final concentration of 30 µg/ml. The plate was incubated at 37 °C, and fluorescence measurements were recorded at three distinct time points (1 h, 14 h, and 24 h post-incubation). Fluorescence intensities were measured at an emission wavelength of 590 nm using a BIOTEK microplate reader. The results are represented as mean ± standard error of six replicates (n=6). Similar to method 6, in an alternate experiment, 100 µl of supernatant from each well was transferred to new 96 well plate to take readings separately.

1. **Cell Viability using MTT Assay:**

Alternative to resazurin assay, we also used conventional MTT assay for confirming the cell viability of LNPs and MLNPs. In this experiment, briefly, A549 cells were seeded at a density of 7,500 cells/well in a 96-well plate and incubated for 24 h to allow cell attachment. Thereafter, LNPs and MLNPs were added at concentrations ranging from 100 – 600 µg/ml, with each well containing a total volume of 200 µl of nanoparticle suspension prepared in complete DMEM high-glucose medium. The plates were then incubated for an additional 24 h. The old medium was replaced with 100 µl of fresh MTT solution prepared in 1X DPBS to achieve a final concentration of 0.5 mg/ml (Stock concentration of MTT: 10 mg/ml). The plate was incubated at 37 °C for 3 h. Upon completion of the incubation, 100 µl of DMSO was administered to each well, and the plate was subjected to orbital agitation for 30 mins to achieve thorough solubilization of the formazan crystals, enabling accurate spectrophotometric quantification. Absorbance was measured at 570 nm using a BIOTEK microplate reader. Data were presented as mean ± standard error of six replicates (n = 6).

1. **MTT Assay for LNPs and MLNPs:**

To assess the direct interaction of MTT with nanoparticles in the absence of cells, a similar protocol was followed. Briefly, the LNPs and MLNPs were aliquoted into a 96-well plate at concentrations equivalent to those used in cell culture experiments, ranging from 100–600 µg/ml, with each well containing a total of 200 µl of nanoparticle suspension in complete DMEM high-glucose medium. Subsequently, after 24 h of incubation, the medium was removed, and MTT solution (stock concentration: 10 mg/ml) prepared in 1X DPBS was added to each well to achieve a final concentration of 0.5 mg/ml. The plate was then incubated at 37 °C for 3 h. Following incubation, 100 µl of DMSO was added to each well. The absorbance was measured at 570 nm using a BIOTEK microplate reader and the results were expressed as mean ± standard error of six replicates (n = 6).

1. **Statistical analysis:**

All samples were analyzed in triplicate for nanoparticle characterization and sextuplicate for the remaining study. Data were presented as mean ± standard error. Statistical significance was assessed using one-way analysis of variance (one-way ANOVA), followed by Dunnett’s post hoc test for multiple comparisons against the control group, with a 95% confidence interval. The observations with a *p*-value less than 0.05 were considered significantly different.

**FIGURES:**


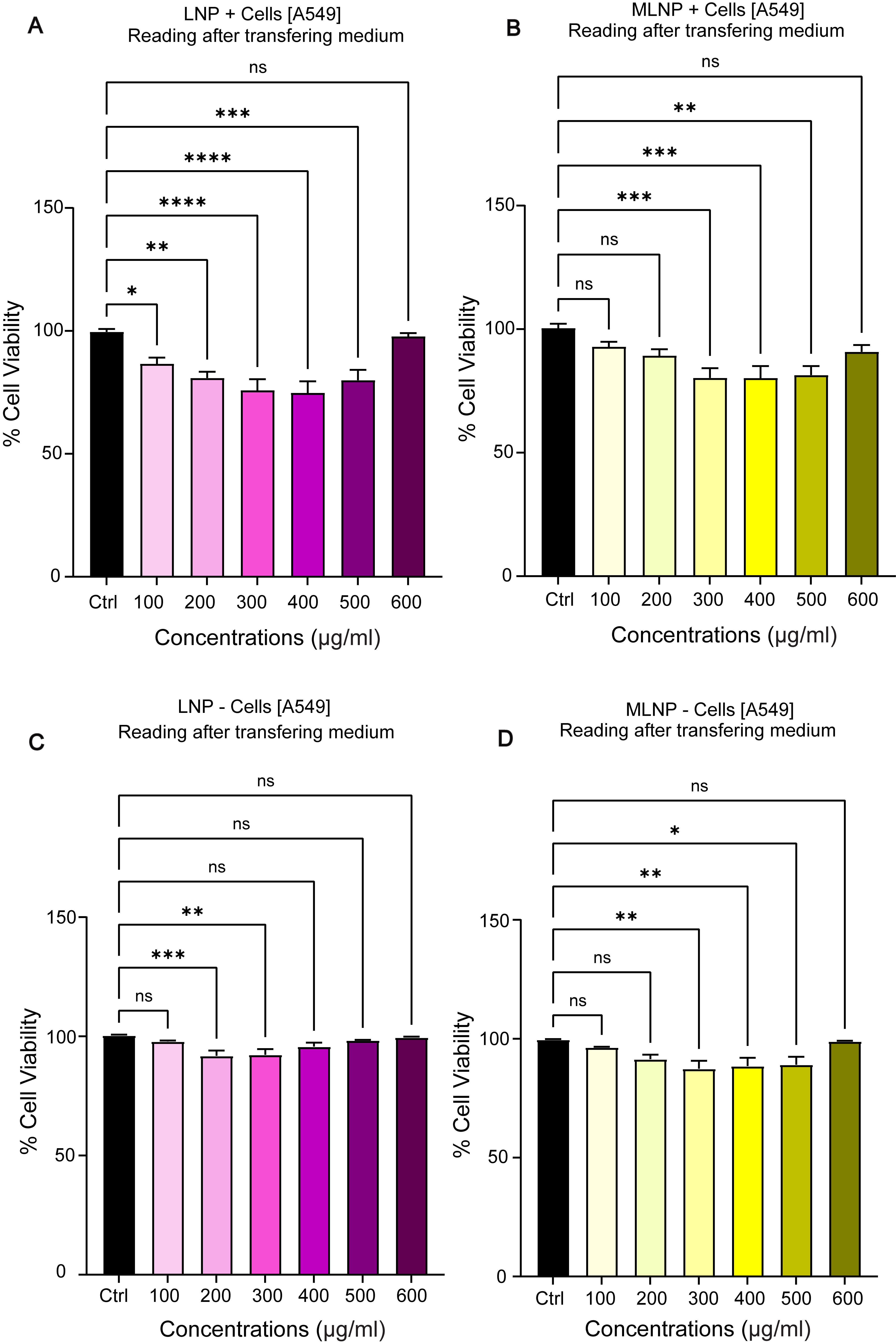


**Figure S1:** **(A and B)** The bar graphs depict viability of A549 cells in presence of LNPs and MLNPs that was determined from supernatant fraction from each well as mentioned in method 6. Data are presented as mean ± standard error of six replicates (n = 6). The asterisks in the bar graphs represent significantly different observations when compared to the control group (One-way ANOVA test,* p < 0.05, ** p < 0.01, *** p < 0.001, **** p < 0.0001). **(C and D)** The bar graphs depict fluorescence intensity of supernatant fraction when resazurin dye was incubated with LNPs and MLNPs in absence of A549 cells as mentioned in method 7. The asterisks in the bar graphs represent significantly different observations when compared to the control group (One-way ANOVA test,* p < 0.05, ** p < 0.01, *** p < 0.001).


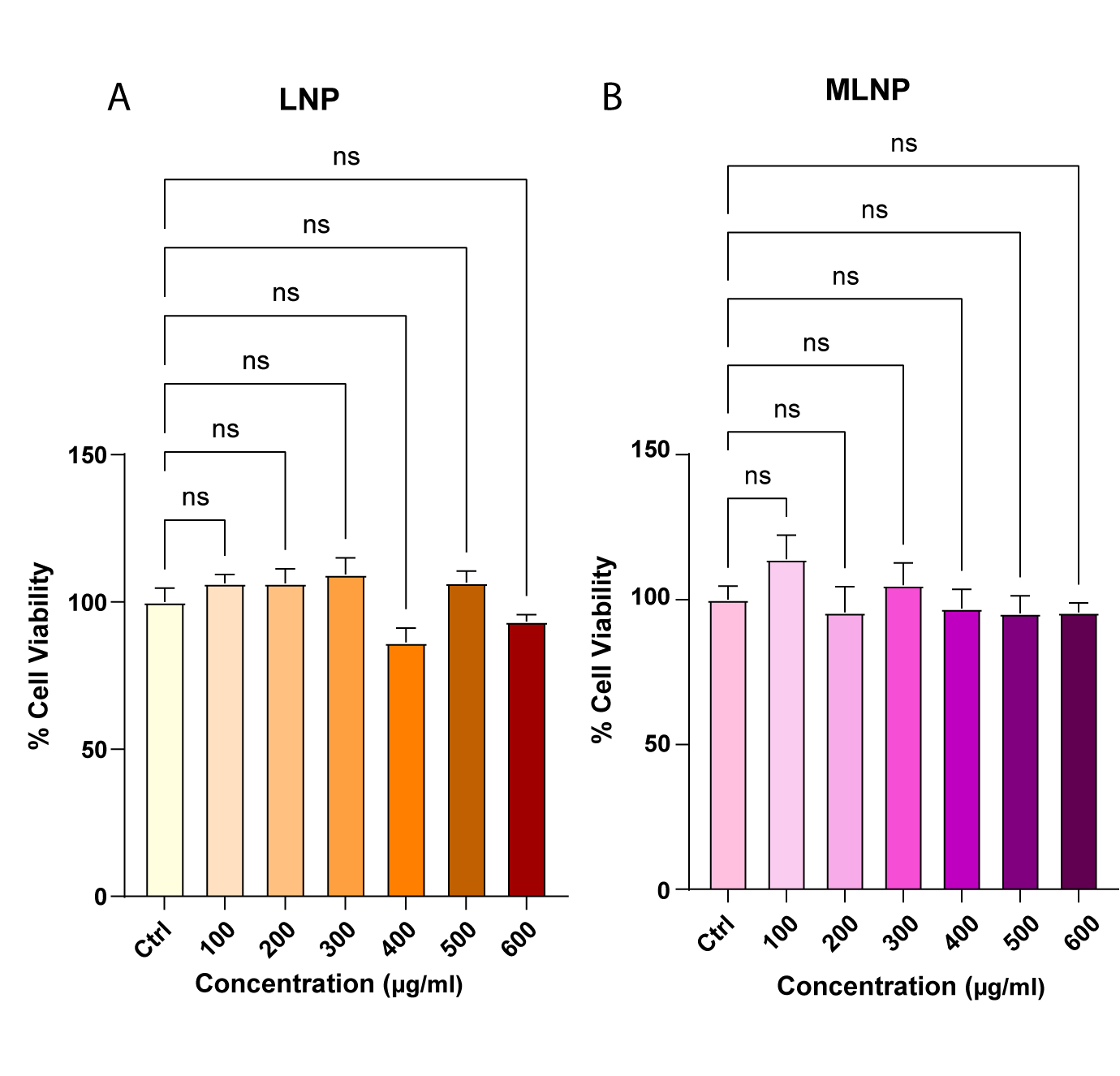


**Figure S2:** **(A and B)** The bar graphs represent the absorbance values obtained from the MTT assay performed for LNPs and MLNPs in the absence of A549 cells as mentioned in the method 9 to assess the direct interaction of MTT with nanoparticles. Data are expressed as mean ± standard error of six replicates (n = 6). Statistical analysis performed using one-way ANOVA indicated no significant differences among treatments as compared to control.

**References:**

1. Chmely S, Gal MR, Arya A, Lin WS, Mendis G. Methacrylated Kraft lignin as a double-edged sword: a quantitative analysis of Kraft lignin-containing objects printed by stereolithography [Internet]. ChemRxiv; 2023 [cited 2025 Apr 10]. Available from: https://chemrxiv.org/engage/chemrxiv/article-details/64d629274a3f7d0c0dfecc77
